# Circulatory shear flow alters the viability and proliferation of circulating colon cancer cells

**DOI:** 10.1038/srep27073

**Published:** 2016-06-03

**Authors:** Rong Fan, Travis Emery, Yongguo Zhang, Yuxuan Xia, Jun Sun, Jiandi Wan

**Affiliations:** 1 Microsystems Engineering, Rochester Institute of Technology, Rochester, New York, USA; 2 Department of Mechanical Engineering, Rochester Institute of Technology, Rochester, New York, USA; 3 Department of Medicine, University of Illinois at Chicago, Chicago, Illinois, USA; 4 Department of Applied Physics and Applied Mathematics/Materials Science and Engineering Program, Columbia University, New York, USA

## Abstract

During cancer metastasis, circulating tumor cells constantly experience hemodynamic shear stress in the circulation. Cellular responses to shear stress including cell viability and proliferation thus play critical roles in cancer metastasis. Here, we developed a microfluidic approach to establish a circulatory microenvironment and studied circulating human colon cancer HCT116 cells in response to a variety of magnitude of shear stress and circulating time. Our results showed that cell viability decreased with the increase of circulating time, but increased with the magnitude of wall shear stress. Proliferation of cells survived from circulation could be maintained when physiologically relevant wall shear stresses were applied. High wall shear stress (60.5 dyne/cm^2^), however, led to decreased cell proliferation at long circulating time (1 h). We further showed that the expression levels of β-catenin and c-myc, proliferation regulators, were significantly enhanced by increasing wall shear stress. The presented study provides a new insight to the roles of circulatory shear stress in cellular responses of circulating tumor cells in a physiologically relevant model, and thus will be of interest for the study of cancer cell mechanosensing and cancer metastasis.

Cancer metastasis, a multistep process in which cancer cells migrate or flow from the primary tumor site to a distal location, causes over 90% of cancer-related deaths^1^,^2^. Over 50% patients with colorectal cancer, for example, develop distant metastasis, making the colorectal cancer the second leading cause of cancer deaths in the United States^2^. During metastasis, circulating tumor cells (CTCs) are transported through the blood circulatory system and are subjected to hemodynamic forces^3^. Although it is known that fluid shear-forces resulted from the bloodstream cause destructions of CTCs and only a small fraction of CTCs can survive and generate metastasis^1^,^4^,^5^, the effect of circulatory shear flow on the viability and proliferation of CTCs remains elusive.

Progress has been made to understand the mechanism of shear stress in the regulation of cancer cells. However, the majority of studies investigate the effects of shear on cells that are immobilized in micro-wells or adhered to microchannels^6^,^7^,^8^. The effect of shear on circulating cancer cells in suspension, however, remains less understood. Approaches, such as cone-and-plate viscometer and stirring bath, have been developed to study the effect of shear on cell suspensions^9^,^10^,^11^. However, the shear conditions are less physiologically relevant, and thus are marginally effective to evaluate the effect of circulatory shear stress on CTCs. Most importantly, previous studies have mainly focused on cell viability after shear stimulation^6^,^9^,^12^, the proliferation of cells that are survived from shear, which plays an important role in the development of secondary tumors, remains unknown.

Here, we developed a microfluidic circulatory system to study the effect of shear stress on the viability and proliferation of circulating HCT116 human colon cancer cells. The microfluidic circulatory platform is a close-loop circulating system that consists of a peristaltic pump, connecting tubing, and a microfluidic channel embedded with a constriction. When HCT116 cells are flowing in the developed microfluidic circulating system, they experience periodically low wall shear stresses in the connecting tubing but increased wall shear stress in the microfluidic channel, mimicking the circulation of CTCs in the blood vascular system composed of large vessels and arterioles. We investigated the viability of HCT116 cells after circulating at different magnitudes of wall shear stress and circulating time. We also examined the proliferation of HCT116 cells that were survived from the circulation and the expression of critical genes related to the β-catenin signaling, a key regulator of cell proliferation. Our study provided a new insight into the effect of circulatory shear stress on circulating tumor cells in a physiologically relevant model.

## Results

### Cell viability decreased at long circulating time but increased at high peristaltic speed

**Figure 1:**
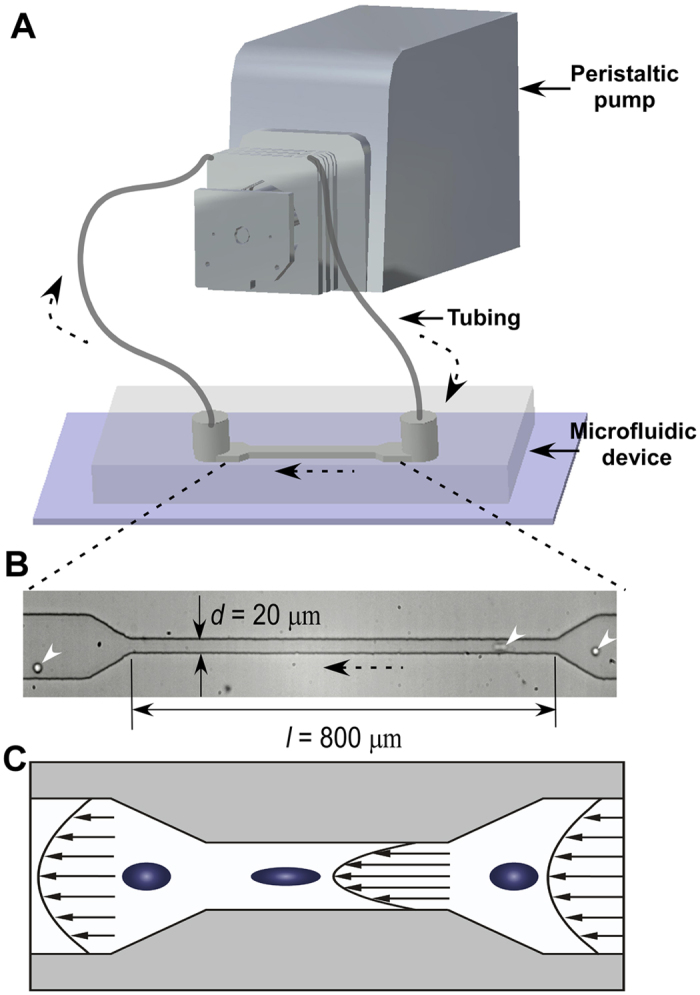
Circulation of HCT116 human colon cancer cells in a circulatory microfluidic platform. (**A**) Schematic of the microfluidic facility for cell circulation. (**B**) A typical microscopic image of cells flowing through the constriction in the microfluidic device. White arrows indicate the cells. Black arrows with dot lines indicate the flow direction. (**C**) Schematic illustration of the elongation of circulating cancer cells when passing through the constriction due to increased shear stress.

We developed a microfluidic system that was composed of a peristaltic pump, silicone tubing, and a microfluidic device with a constriction channel to study the effect of periodic circulatory shear stress on circulating HCT116 colon cancer cells (Fig. 1A). Wall shear stress was calculated based on the peristaltic speed of circulation and the geometry of microfluidic channel (Table S1). Particularly, cells circulating at 0.1 revolution per minute (rpm) were subjected to a wall shear stress of approximate 3.5 dyne/cm^2^ at the constriction and 0.46 dyne/cm^2^ in the wide channel (Fig. 1B and C), which were close to the physiologically relevant shear conditions in venous circulation (0.5–4 dyne/cm^2^)^13^. Circulation at 0.5 rpm provided a wall shear stress of 26.9 and 3.56 dyne/cm^2^ for the constriction and wide channel respectively, close to the wall shear stress in arterial circulation (4–30 dyne/cm^2^)^13^. The speed of 1.0 rpm, generated relatively high wall shear stresses at both of the constriction (60.5 dyne/cm^2^) and wide channel (8 dyne/cm^2^). It should be noted that due to the complex pulsatile three-dimensional flow in the microfluidic circulatory system, quantitative identification of local shear stresses near single circulating cells is experimentally challenging^14^. The calculated wall shear stress thus represents approximate average shear values in flow and cannot be treated as definitive shear stress experienced by single cells.

**Figure 2:**
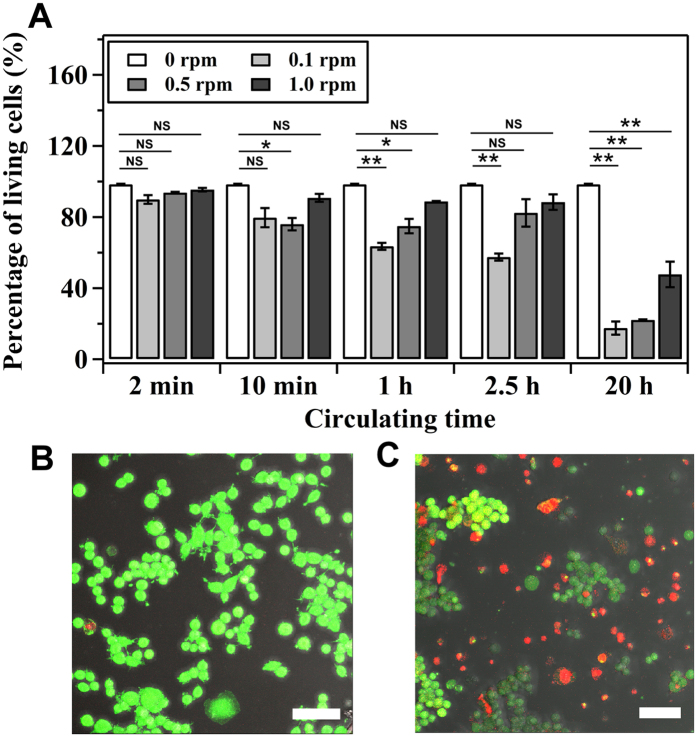
Effect of shear on the viability of circulating HCT116 cells. (**A**) Percentage of living cells that were circulating for 2 min, 10 min, 1 h, 2.5 h and 20 h in the microfluidic circulatory system at a speed of 0, 0.1, 0.5 or 1.0 revolution per minute (rpm). **P < 0.01 and *P < 0.05 were calculated based on paired student *t*-test analysis. NS = non-significant. (**B**,**C**) is the fluorescent image of cells stained with Live/Dead assay after circulation at flow rate of 1.0 rpm for 2 min and 20 h, respectively. Scale bar: 50 μm.

To study the effect of shear and circulating time on the viability of HCT116 colon cancer cells, we circulated the cells in the microfluidic circulatory system at different peristaltic speeds (rpm) and circulating time. The results showed that, at a constant peristaltic speed, cell viability decreased with the increase of circulating time (Fig. 2A). 2 min circulation at 1.0 rpm, for example, barely affected the cell viability (Fig. 2B), whereas significant cell death was found when cells were circulated for 20 h (Fig. 2C). The results are consistent with previous studies in which cell viability decreases with increased duration of applied shear^12^,^15^. On the other hand, when the circulating time was kept constant, the effect of peristaltic speed on cell viability (comparing to control) was not significant for circulating time of 2 min and 10 min (*P* > 0.05, Student’s *t*-test, except rpm = 0.5 for circulating time 10 min). When the circulating time increased to 1 h, 2.5 h and 20 h, however, cell viability increased with the increase of peristaltic speed.

### Proliferation of survived cells decreased at increased circulating time and peristaltic speed

**Figure 3:**
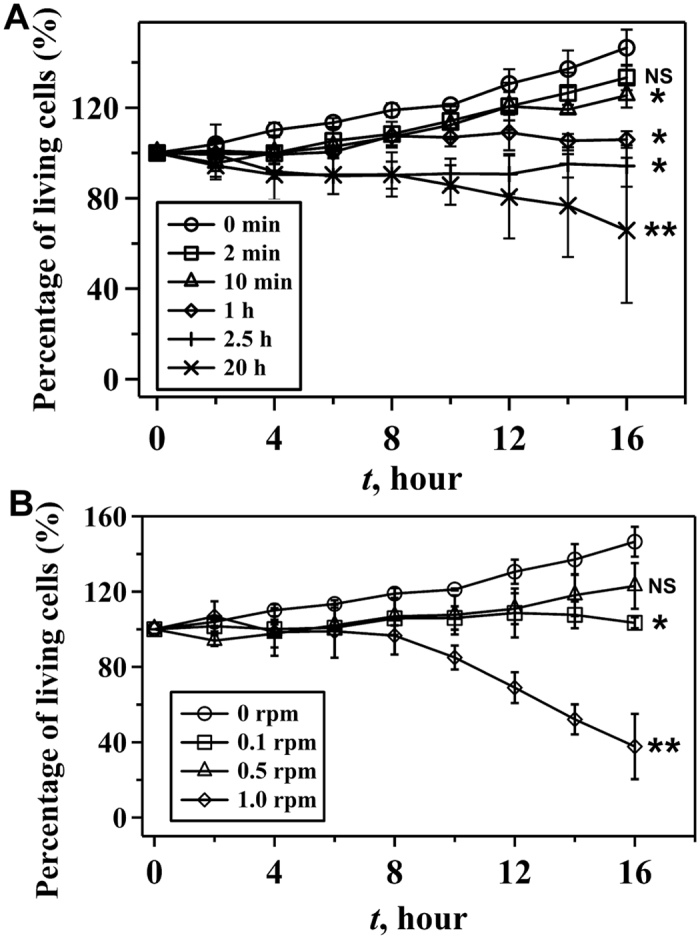
Effect of shear on the proliferation of survived HCT116 cells after circulation. (**A**) Percentage of living cells at different culture time after circulation at 0.1 rpm for 0 min, 2 min, 10 min, 1 h, 2.5 h and 20 h. (**B**) Percentage of living cells at different culture time after circulation at the speed of 0, 0.1, 0.5 and 1.0 rpm for 1 h. **P < 0.01 and *P < 0.05 were calculated based on paired student *t*-test analysis. NS = non-significant.

Next, we cultured cells that were survived from the microfluidic circulatory system for 16 hours and monitored the cell growth. The effect of circulating time on cell growth was studied using cells that were circulated at a constant peristaltic speed (0.1 rpm) (Fig. 3A). Comparing to the viability of control cells (145%), viability of cells circulated for 2 min and 10 min was not significantly different with control (cell viability was 133% and 125% for cells circulated for 2 and 10 min respectively, *P* > 0.05). The results suggested that, for a short duration of applied shear stress, cell proliferation was not compromised significantly. Cells experienced 1 h and 2.5 h circulation showed the viability of 106% and 94% respectively, indicating that cell proliferation was affected by the circulation. Long-term circulation (20 h), however, resulted in a significant (*P* * <*  0.01) decrease of cell viability (65%).

When the circulating time was constant (1 h), the proliferation of survived cells was also negatively affected by the peristaltic speed. The percentages of living cells after 16 h culture, for example, were 106% and 123% for cells treated with 0.1 and 0.5 rpm respectively (Fig. 3B). Significant (*P* < 0.01) decrease of the cell proliferation (38%) was observed when cells were treated with 1.0 rpm circulation. Thus, cell proliferation decreased with the increasing peristaltic speed, although high cell viability at increased peristaltic speed was observed in Fig. 2A.

### Expression of critical genes related to β-catenin signaling was up-regulated by circulation

**Figure 4:**
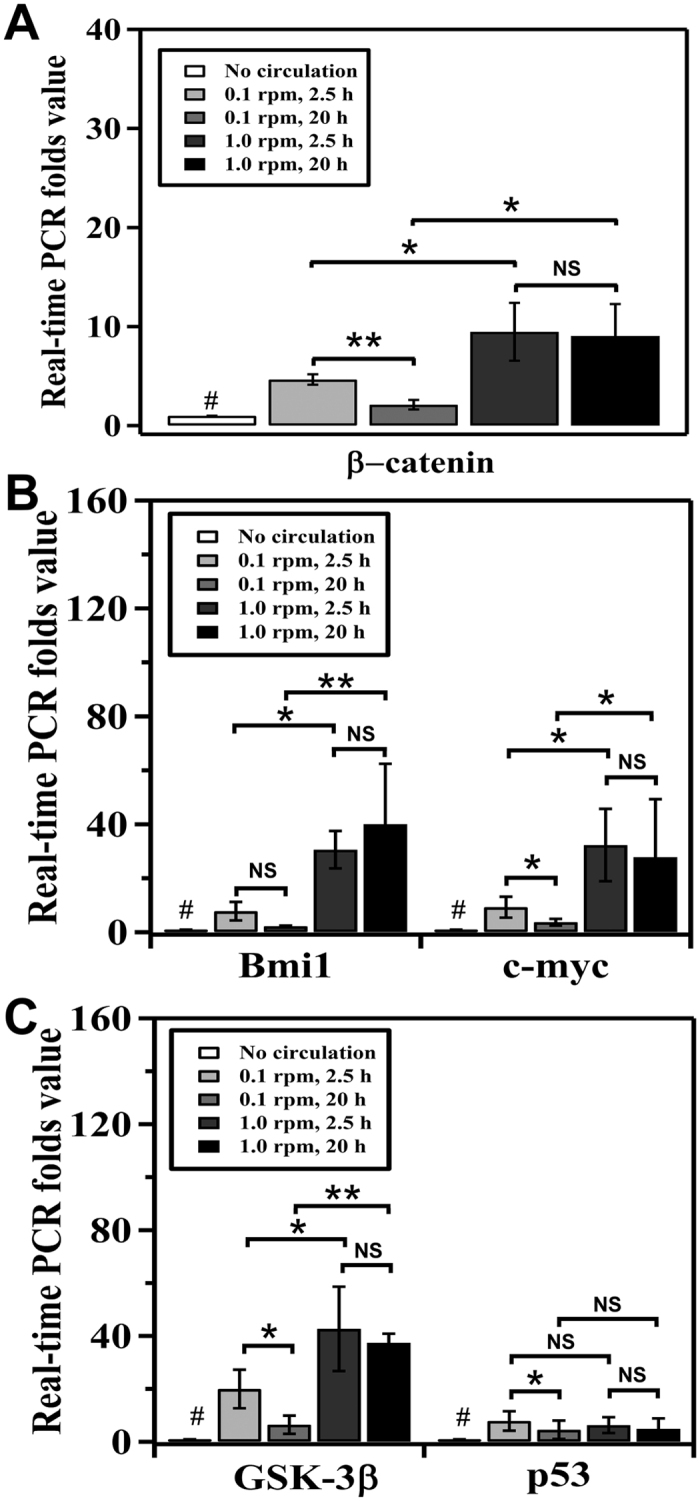
Effect of shear on the mRNA expression level of (**A**) β-catenin, (**B**) Bmi1 and c-myc, and (**C**) GSK-3β and p53 mRNA in HCT116 cells circulated at speeds of 0.1 and 1.0 rpm for 2.5 h and 20 h respectively. **P < 0.01 and *P < 0.05 were calculated based on paired student t-test analysis. ^#^*P* < 0.05 was calculated, individually, between control (no circulation) and the rest of the sample. NS = non-significant.

Because β-catenin plays significant roles in cell cycle, proliferation and apoptosis^16^, we further studied the expression of β-catenin at the mRNA level in response to shear stimulation (Fig. 4A). The real-time PCR results showed that, comparing to control, shear stress always induced increased mRNA expression of β-catenin. Long time (20 h) circulation at constant wall shear stresses decreased or did not affect significantly the expression of β-catenin comparing to that of short term circulation (2.5 h). At a constant circulation time, β-catenin expression increased with the increase of the magnitude of wall shear stress. The increased expression of β-catenin with shear was opposite to previous studies where β-catenin/Wnt signaling pathway was inhibited by shear stress when cells were immobilized on surfaces^11^,^17^.

To further understand the effects and regulation of β-catenin expression, we measured the expression of Bmi1, c-myc, and glycogen synthase kinase 3β (GSK-3β), as the regulator of β-catenin’s stability. Our results showed that significant (*P* < 0.05) increase of expression of both Bmi1 and c-myc was observed at high wall shear stress (Fig. 4B). Because Bmi1 and c-myc were known as the downstream targets of the β-catenin signaling pathway, increased expression of β-catenin led to the increased expression of both Bmi1 and c-myc at high magnitude of wall shear stress. On the other hand, GSK-3β is responsible for the degradation of β-catenin and negatively regulates β-catenin expression^18^. In our experiments, expression of GSK-3β was (*P* < 0.05) promoted by high wall shear stress. P53 is also known to regulate cell apoptosis, proliferation and responses to stresses^19^. However, no significant (*P* > 0.05) effect of high shear on the expression of p53 was observed (Fig. 4C).

## Discussion

Although the influence of shear stress on the growth of adhered colon cancer cells has been reported previously^6^,^7^,^8^, the effect of shear stress on circulating colon cancer cells remains less understood. Here, we demonstrated a microfluidic circulatory system to closely mimic the physiologically relevant shear conditions in circulation and showed that the viability, proliferation, and gene expression of circulating HCT116 cells depended significantly on the circulatory wall shear stress and circulating time.

We showed that when wall shear stress was kept constant, cell viability decreased with increasing circulating time (Fig. 2A). The results were consistent with previous studies on other cell lines^20^,^21^ and can be explained by the observed expression of β-catenin in Fig. 4A, in which the expression of β-catenin decreased with increased circulating time (from 2.5 h to 20 h). Remarkably, HCT116 colon cancer cells had high cell viability when experiencing high magnitude of wall shear stress (Fig. 2A) and the expression of β-catenin was also increased with shear (Fig. 4A).

The results of increased cell viability and β-catenin expression at high shear, however, are surprising because previous studies showed that the viability of cells (such as SW480, HT29, and SW620 colon cancer cells) decreased with increased shear and β-catenin expression was inhibited upon shear stimulation^17^,^22^,^23^,^24^,^25^,^26^,^27^. These studies, however, were based on cells that were adhered on surfaces and, thus, the shear condition in which cells were experienced was different with our current setup. This may cause different regulation mechanisms in terms of intracellular signaling. During circulation in suspension, for example, the role of adhesive molecule, such as α6β4 integrin that was promoted by shear flow in adhered SW620 colon cancer cells^17^,^23^, became not significant. Because α6β4 was able to activate the expression of p53 that enhanced the degradation of β-catenin, the non-activated expression of p53 due to the lack of activation of α6β4 would not be able to degrade β-catenin^28^. Indeed, our data showed that p53 expression was not significantly affected by wall shear stress (Fig. 4C). We speculate that β-catenin expression was not compromised by shear stimulation in our experiments (Fig. 4A).

In addition, the circulation in our microfluidic system generated a periodic non-constant shear stress, which was known to be able to affect cellular behavior and intracellular signaling differently, compared to constant laminar shear stress^29^,^30^,^31^. From the investigation of osteocytic cells, for example, periodic shear stress was found to be able to activate the secretion of Prostaglandin E_2_ (PGE2) in a more effective manner than constant flow^32^. Enhancement of PGE2 signaling could then promote the Akt-mediated phosphorylation of GSK-3β, improving the translocation of β-catenin to nucleus. In addition, when human umbilical vein endothelial cells were treated by periodic flow conditions, the vasodilator nitric oxide synthase mRNA was found to be significantly up-regulated comparing with steady shear stress at the same magnitude^33^. Therefore, it was also possible that the expression of β-catenin in HCT116 colon cancer cells preferred a periodic non-constant shear condition to a constant flow. In fact, we showed an increased expression of Bmi1 and c-myc at high circulatory shear stress that was known to enhance the nucleus translocation of β-catenin. However, it should be noted that although we observed high cell viability at high shear, proliferation of survived cell decreased with the increase of shear, suggesting that intracellular functions regulated by the β-catenin signaling pathway were significantly disrupted by the shear stimulation. Note that concentration of circulating cells did not affect significantly cell viability immediately after circulation but had an influence on the proliferation of survived cells (Fig. S1). Nevertheless, HCT116 cells were still able to survive in circulation at 0.1 and 0.5 rpm that had physiologically relevant shear conditions (wall shear stress ≈4–30 dyne/cm^2^) for at least 1 hour and proliferate. The underlying mechanisms remain to be determined.

In summary, we have developed a microfluidic system to study the effect of periodic circulatory shear stress on circulating HCT116 cells. Cell viability decreased at long circulating time but increased at high magnitude of wall shear stress. We showed that the expression of β-catenin increased at high wall shear stress. The increased expression of β-catenin and cell viability was probably due to the circulatory shear conditions that could lead to different intracellular signaling pathways comparing to constant laminar shear stress experienced by adhered cells. Proliferation of cells survived from circulation could be maintained when cells were circulated at physiologically relevant conditions, but decreased at increasing wall shear stress and circulating time. The current study thus revealed a previously unrecognized role of circulatory shear stress on CTCs and contributed to the acquaintance of shear stimulation on CTCs *in vivo*. In addition, the developed microfluidic approach that could create physiologically relevant microenvironments of CTCs in circulation offered potential techniques to develop model systems for the study of attachment and invasion of CTCs during circulation and cancer metastasis.

## Methods

### Cell maintenance

Human colon cancer HCT 116 cells were cultured in a T-25 culture flask supplied with DMEM (Life Technologies) containing 10% (v/v) fetal bovine serum (FBS) (Life Technologies) and 1% (v/v) penicillin/streptomycin (Life Technologies) under 37 °C and 5% CO_2_. To prepare the cell suspension, cells were washed by a PBS solution (Life Technologies) and treated with Trypsin (Life Technologies) for 5 min to detach the cell from the culture flask. The cell suspension was then centrifuged and re-suspended in a fresh DMEM supplied with 10% FBS in a concentration of 1 × 10^8^/ml. Because dead cells did not attach on the surface of the culture flask and were washed away by PBS, the viability of cells in the suspension was approximately the same for all experiments.

### Microfluidic fabrication and circulation of HCT116 colon cancer cells

The microfluidic device was fabricated by using the standard soft lithography technique in poly(dimethysiloxane) (PDMS) (Sylgard 184, Dow Corning). The microfluidic device contains a wide straight channel with 100 μm width and a constriction channel with 800 μm length and 20 μm width (Fig. 1), comparable to the typical size of arterioles^34^. The height of the microchannel is 37 μm everywhere. Two polyethylene tubing (Scientific Commodities Inc, 0.015″ (0.38 mm) I.D. × 0.043″ (1.09 mm) O.D.) were inserted into the inlet and outlet of the microfluidic device, respectively. A large silicone tubing (Tygon, 0.031″ I.D. × 0.094″ O.D.) was connected to the ends of polyethylene tubing through adaptors (Qosina) to establish a close-loop circulatory system (Fig. 1). Last, the silicone tubing was wrapped on the rollers of a peristaltic pump that drove the circulation by squeezing the silicone tubing via rotating rollers.

All tubing and microfluidic devices were sterilized by 70% ethanol (Sigma) for 10 min and washed by PBS for all experiments. Air bubbles in the microfluidic system were removed during the washing steps using ethanol and PBS. The peristaltic pump was sterilized and placed into the incubator with the microfluidic device. No specific channel treatment was conducted in the experiment. 1.5 mL HCT116 cell suspension (0.01, 1, or 100 × 10^8^ cells/ml) was injected into the microfluidic circulatory system through the polyethylene tubing connected with the inlet of the microfluidic device. The flow rate of circulation was controlled by changing the rotating speed (revolution per minute (rpm)) of the roller in the peristaltic pump. A peristaltic speed of 0.1, 0.5 or 1.0 rpm was used.

### Determination of cell viability and growth after circulation

To analyze cell viability immediately after circulation, we collected cells from the microfluidic circulatory system and allowed cells to sediment to the surface of a petri dish (~1 hour). Cell viability was then determined by using the Live/Dead assays (Life Technologies) following the manufacture’s protocol. Images of cells stained with Live/Dead assays were analyzed using a confocal microscope (Leica Microscope, SP5). To monitor the growth of cells that were survived after circulation, cells were collected in a petri dish supplied with a DMEM culture medium and allowed to settle for 2 hours in an on-stage incubator with 37 °C and 5% CO_2_. Cells that were survived after circulation could adhere on the surface of the petri dish. The growth of survived cells was recorded by a camera (C10600-10B-H, Hamamatsu) mounted on a fluorescence microscope (Leica Microsystems, DMI 6000) for 16 hours. Time-lapse videos of adhered cells were used to analyze the rate of cell growth. The number of cells was measured every 2 hours.

### Quantitative real-time PCR

Total RNA was extracted from circulated cells using TRIzol reagent (Invitrogen, Grand Island, NY, USA). The RNA integrity was verified by electrophoresis. RNA reverse transcription was performed using the iScript cDNA synthesis kit (Bio-Rad, Hercules, CA, USA) according to the manufacturer’s protocol. The RT cDNA reaction products were subjected to quantitative real-time PCR using CTFX 96 Real-time system (Bio-Rad, Hercules, CA, USA) and SYBR green supermix (Bio-Rad, Hercules, CA, USA) according to the manufacturer’s protocol. The primers are listed in Table S2. All expression levels were normalized to β-actin levels of the same sample. Percent expression was calculated as the ratio of the normalized value of each sample to that of the corresponding untreated control cells. All real-time PCR reactions were performed in triplicate.

### Statistical analysis

Each set of the experiment was repeated for at least three times with more than 100 cells being measured. The error bar was presented as the standard deviation of the mean for all trials. Data sets were plotted using the software of Igor Pro (Wave Metrics, Inc). A two-tailed paired *t*-test was used for the analysis of cell viability, cell proliferation and PCR results. The comparisons between two groups with *P* < 0.05 are considered significant.

## Additional Information

**How to cite this article**: Fan, R. *et al*. Circulatory shear flow alters the viability and proliferation of circulating colon cancer cells. *Sci. Rep.* **6**, 27073; doi: 10.1038/srep27073 (2016).

## Supplementary Material

Supplementary Information
