# Supplementary material for: Circulatory shear flow alters the viability and proliferation of circulating colon cancer cells
Source: Sci Rep. 2016 Jun 3;6:27073. doi: 10.1038/srep27073 (PMC4891768; doi:10.1038/srep27073)
Supplement: Supplementary Information [file srep27073-s1.pdf]

Support Information

Circulatory shear flow alters the viability and proliferation of circulating colon cancer cells

Rong Fan,[a] Travis Emery,[b] Yongguo Zhang,[c] Yuxuan Xia,[d] Jun Sun,[c]* Jiandi Wan[a]*

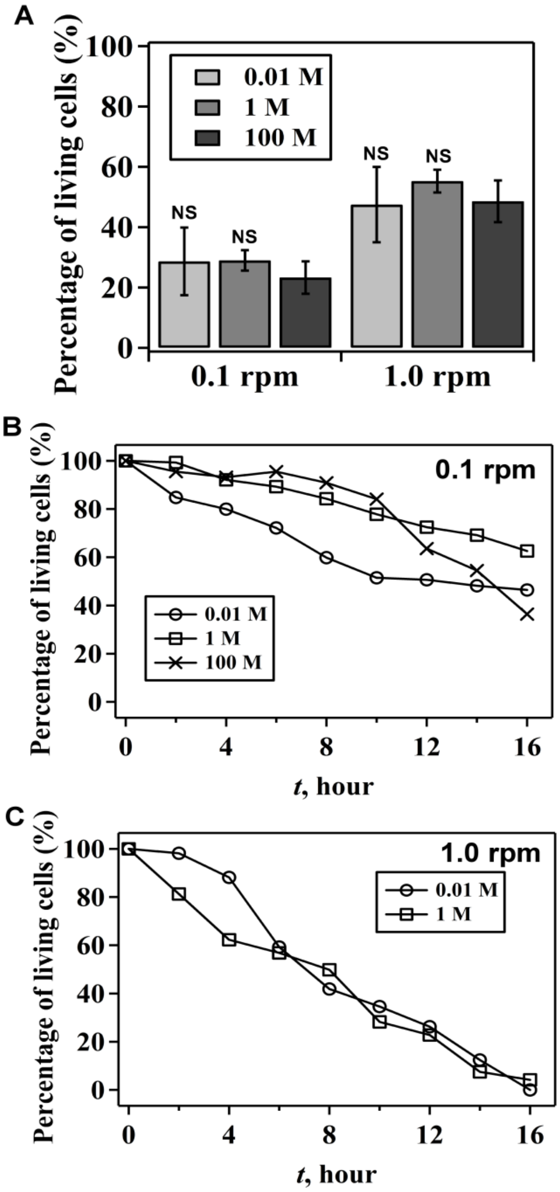

Figure S1. Effect of concentration of circulation cells on cell viability and proliferation. (A) Immediate cell viability after cells circulated for 20 h with an initial cell concentration of 0.01, 1, or 100 M cells/ml. The circulation speeds were 0.1 and 1.0 revolution per minute (rpm). (B) and (C) are proliferation of cells survived from 20 h circulation at 0.1 rpm and 1.0 rpm respectively. The initial cell concentration was 0.01, 1, or 100 M cells/ml. Note the different proliferation of cells in (B) at different initial cell concentrations. ** $P < 0.01$ and * $P < 0.05$ were calculated based on paired student *t*-test analysis. NS = non-significant.

Table S1. Calculation of average wall shear stress in the microfluidic system

**Calculating parameter**	**Value**
Viscosity (μ)	$1 \times 10^{-3} \mathrm{Pa \cdot s}$
Volume flow rate (*Q*) for 0.1 rpm	$2.5 \times 10^{-10}\ \mathrm{m^3/min}$
Volume flow rate (*Q*) for 0.5 rpm	$1.9 \times 10^{-9}\ \mathrm{m^3/min}$
Volume flow rate (*Q*) for 1.0 rpm	$4.3 \times 10^{-9}\ \mathrm{m^3/min}$
Width of the constriction channel (*w*)	$20 \times 10^{-6}\ \mathrm{m}$
Height of the constriction channel (*h*)	$30 \times 10^{-6}\ \mathrm{m}$
Width of the wide microchannel (*w*)	$100 \times 10^{-6}\ \mathrm{m}$
Height of the wide microchannel (*h*)	$30 \times 10^{-6}\ \mathrm{m}$
Inner diameter of the tubing (*d*)	$3.8 \times 10^{-4}\ \mathrm{m}$
Approximate average shear stress in the constriction channel (τ_c)	
0.1 rpm	$3.5\ \mathrm{dyn/cm^2}$
0.5 rpm	$26.9\ \mathrm{dyn/cm^2}$
1.0 rpm	$60.5\ \mathrm{dyn/cm^2}$
Approximate average shear stress in the wide microchannel (τ_w)	
0.1 rpm	$0.46\ \mathrm{dyn/cm^2}$
0.5 rpm	$3.56\ \mathrm{dyn/cm^2}$
1.0 rpm	$8\ \mathrm{dyn/cm^2}$
Approximate average shear stress in the tubing (τ_t)	
0.1 rpm	$0.0077\ \mathrm{dyn/cm^2}$
0.5 rpm	$0.06\ \mathrm{dyn/cm^2}$
1.0 rpm	$0.13\ \mathrm{dyn/cm^2}$

Note that τ_c and τ_w are calculated based on the equation $\tau = \frac{\mu Q}{w^2 \times h}$, whereas τ_t is calculated based on the equation $\tau = \frac{32\ \mu Q}{\pi d^3}$.

Table S2. Primers for real-time PCR

Gene name	Primers
β-actin F	AGAGCAAGAGAGGCATCCTC
β-actin R	CTCAAACATGATCTGGGTCA
β-catenin F	AAAATGGCAGTGCGTTTAG
β-catenin R	TTTGAAGGCAGTCTGTCGTA
Bmi 1 F	AGCAGAAATGCATCGAACAA
Bmi 1 R	CCTAACCAGATGAAGTTGCTGA
c-myc F	ACAGCTACGGAACTCTTGTGC
c-myc R	GCCCAAAGTCCAATTTGAGGC
GSK-3β F	GGAACTCCAACAAGGGAGCA
GSK-3β R	TTCGGGGTCGGAAGACCTTA
P53 F	CCCAAGCAATGGATGATTTGA
P53 R	GGCATTCTGGGAGCTTCATCT
